# Automated insulin delivery in older adults with type 1 diabetes: evidence, limitations, and clinical perspectives

**DOI:** 10.1007/s12020-026-04816-z

**Published:** 2026-10-29

**Authors:** Francesco Galeano, Agnese Rita Catena Mobilia, Filippo Luca Fimognari, Vittorio Oteri, Rossella Cannarella, Giuseppe Papa, Rosario Le Moli, Tommaso Piticchio, Antonino Di Pino, Maurizio Di Marco, Sabrina Scilletta, Francesco Frasca, Andrea Tumminia

**Affiliations:** 1 Department of Clinical and Experimental Medicine, Endocrinology Section, Garibaldi-Nesima Hospital, University of Catania, Catania, 95122 CT Italy; 2 Department of Medicine, Unit of Geriatrics, Azienda Ospedaliera “Annunziata - Mariano Santo - S. Barbara”, Cosenza, 87100 CS Italy; 3 Department of Clinical and Experimental Medicine, University of Catania, Catania, Italy; 4 Unit of Metabolic and Endocrine Disease, “Centro Catanese di Medicina e Chirurgia” Clinic, Catania, Italy; 5 Department of Medicine and Surgery, University Kore of Enna, Enna, Italy; 6 Unit of Diabetology, Metabolic and Endocrine Diseases, “Cannizzaro” Emergency Hospital, Catania, Italy; 7 Department of Clinical and Experimental Medicine, Internal Medicine, Garibaldi- Nesima Hospital, University of Catania, Catania, 95122 CT Italy; 8 Endocrine Unit, Garibaldi-Nesima Hospital, Catania, 95122 CT Italy

**Keywords:** Type 1 diabetes, Older adults, Automated insulin delivery, Hybrid closed-loop, Hypoglycemia, Diabetes technology.

## Abstract

**Objective:**

Automated insulin delivery (AID) systems have significantly improved the management of type 1 diabetes (T1D), leading to better glycemic control and reduced hypoglycemia. However, older adults (≥ 65 years) remain underrepresented in clinical trials, and evidence specific to this population is limited.

**Design and patients:**

This narrative review synthesizes current evidence on the efficacy, safety, and usability of AID systems in older adults (≥ 65 years) with T1D. A comprehensive literature search of MEDLINE/PubMed was conducted using the PICO framework to identify relevant RCTs, observational studies, and systematic reviews, supplemented by literature studies on cognitive, functional, and psychosocial factors impacting technology adoption.

**Results:**

While data from randomized trials and meta-analyses in the general population consistently demonstrate improvements in time in range (TIR, 70–180 mg/dL) and reductions in hypoglycemia, evidence in older adults is still emerging. Frail individuals and those with multiple comorbidities are often underrepresented or excluded from current studies. Nevertheless, the available evidence suggests that many of these benefits are also maintained in older populations, particularly regarding hypoglycemia reduction. At the same time, factors such as cognitive impairment, functional limitations, caregiver dependence, and reduced digital literacy may affect the adoption and effective use of these technologies.

**Conclusions:**

AID systems appear effective and generally safe in selected older adults, with benefits in glycemic control and hypoglycemia reduction. However, given the marked heterogeneity of this population, further studies are needed to better define their role and applicability in routine clinical practice.

**Graphical Abstract:**

**Figure:**
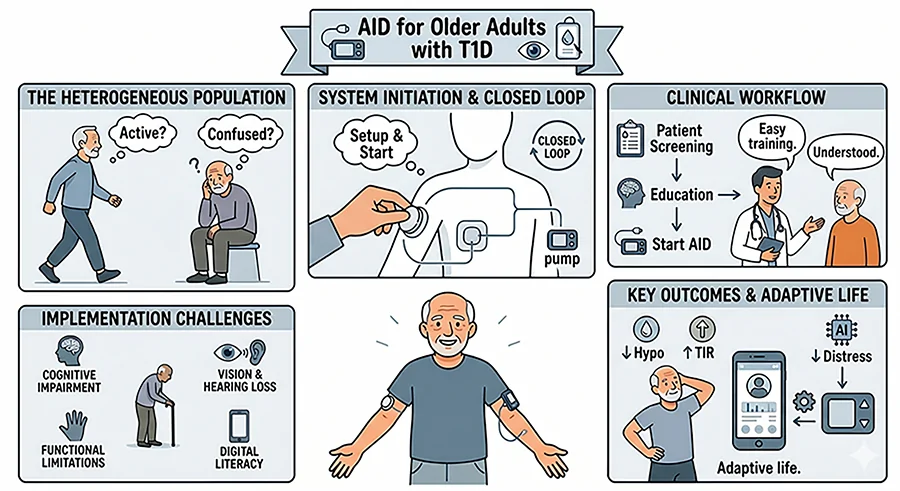

## Introduction

The increasing life expectancy of individuals with type 1 diabetes (T1D) has led to a growing population of older adults living with this condition. Improvements in insulin formulations, glucose monitoring technologies, structured diabetes education, and multidisciplinary care have substantially reduced mortality and delayed the onset of chronic complications, allowing many individuals with T1D to reach older age [1]. Consequently, clinicians are increasingly confronted with the complex management of older adults with long-standing T1D, a population characterized by marked clinical heterogeneity and specific therapeutic challenges [2]. Older individuals frequently present with multiple comorbidities and geriatric syndromes, including cardiovascular disease, chronic kidney disease, visual impairment, neuropathy, sarcopenia, falls, disability, polypharmacy, malnutrition, and depression, often accompanied by functional dependence and varying degrees of cognitive dysfunction [1, 2]. An additional underappreciated issue is the misdiagnosis of T1D as type 2 diabetes (T2D) in older adults, which may lead to inappropriate therapeutic decisions, including unintended discontinuation of insulin therapy [3]. These factors may significantly impair self-management abilities and adherence to intensive insulin therapy, requiring a personalized and multidimensional approach to care that balances glycemic control with safety, preservation of autonomy, and quality of life [4].

The concept of frailty has emerged as particularly relevant in the aging T1D population. Frailty is defined as a state of increased vulnerability resulting from age-related decline in physiological reserve and resilience to stressors [5]. Importantly, in people with T1D older age and lower BMI are the main determinants of muscle decline [6]. Beyond representing age-related comorbidities, frailty and sarcopenia may directly influence glycemic instability and insulin requirements. Reduced skeletal muscle mass, decreased physical activity, malnutrition, and altered nutritional intake may contribute to increased glucose variability and unpredictable insulin sensitivity in older adults with T1D. These factors may further complicate the performance and day-to-day management of AID systems, particularly in clinically vulnerable individuals.

In individuals with diabetes, frailty is associated with higher risks of disability, hospitalization, institutionalization, and mortality. Cognitive decline and dementia are also increasingly recognized in older adults with T1D. The ADA Standards of Care 2026 recommend annual screening for mild cognitive impairment or dementia in adults aged ≥ 65 years (Recommendation 13.3, Level B) and recognize diabetes-related dementia, as a clinical entity characterized by slower progression, absence of typical neuroimaging findings, advanced age, elevated HbA1c levels, long diabetes duration, high frequency of insulin use, frailty, sarcopenia, and dynapenia [2]. These conditions may severely impair the ability to perform complex diabetes self-management tasks, such as carbohydrate counting, insulin dose calculation, and interpretation of glucose trends.

Technological advances have profoundly transformed T1D management. Automated insulin delivery (AID) systems, also referred to as hybrid closed-loop (HCL) or advanced hybrid closed-loop (AHCL) systems, combine continuous glucose monitoring (CGM), insulin pumps, and sophisticated control algorithms capable of dynamically adjusting insulin delivery based on real-time glucose data [7].

Clinical trials evaluating AID systems have consistently shown improvements in time in range (TIR, 70–180 mg/dL), reductions in HbA1c, decreased exposure to hypoglycemia, and improved nocturnal glycemic control compared with conventional insulin pump therapy or multiple daily injections [7–9].

Despite these promising developments, older adults remain markedly underrepresented in pivotal AID trials, which often excluded participants above certain age thresholds or those with significant comorbidities, cognitive impairment, or frailty [10]. Nevertheless, available data suggests that older adults can benefit from AID, with reports of improved outcomes, high treatment satisfaction and sustained device use even in advanced age groups, challenging the assumption that older patients are unable or unwilling to adopt such technologies [11, 12].

This narrative review aims to critically evaluate the available evidence on the use of AID systems in older adults with T1D, focusing on efficacy, safety and usability, while highlighting current knowledge gaps and future research priorities in this rapidly evolving field.

## Methods

This narrative review searched the MEDLINE database via PubMed up to the 3rd of June 2026, combining terms and appropriate indexing vocabulary referring to the following topics: type 1 diabetes, automated insulin delivery, closed-loop, hybrid closed-loop, older adults, frailty and cognitive decline.

The reference lists of the included articles and previous literature reviews on the topic were reviewed for further identification of potentially relevant studies.

Eligible studies included those investigating AID systems, with particular attention to studies including participants aged ≥ 65 years or reporting age-stratified outcomes selected according to relevance and study quality.

Because AID use in older adults is intrinsically linked to geriatric syndromes, literature addressing cognitive impairment, frailty, sarcopenia, and functional/psychosocial factors was intentionally included. The rationale for incorporating these non-AID-specific studies is to provide the clinical and physiological context necessary to understand how age-related vulnerabilities affect automated insulin delivery performance, usability, and safety.

Furthermore, given the historical underrepresentation of adults aged ≥ 65 years in pivotal diabetes technology trials, selected high-quality studies and meta-analyses encompassing broader adult T1D populations (including participants < 65 years) were included. These studies provide foundational context regarding general AID mechanisms and overall glycemic efficacy, from which potential benefits and safety profiles in older adults are contextualized and critically extrapolated.

**Fig. 1:**
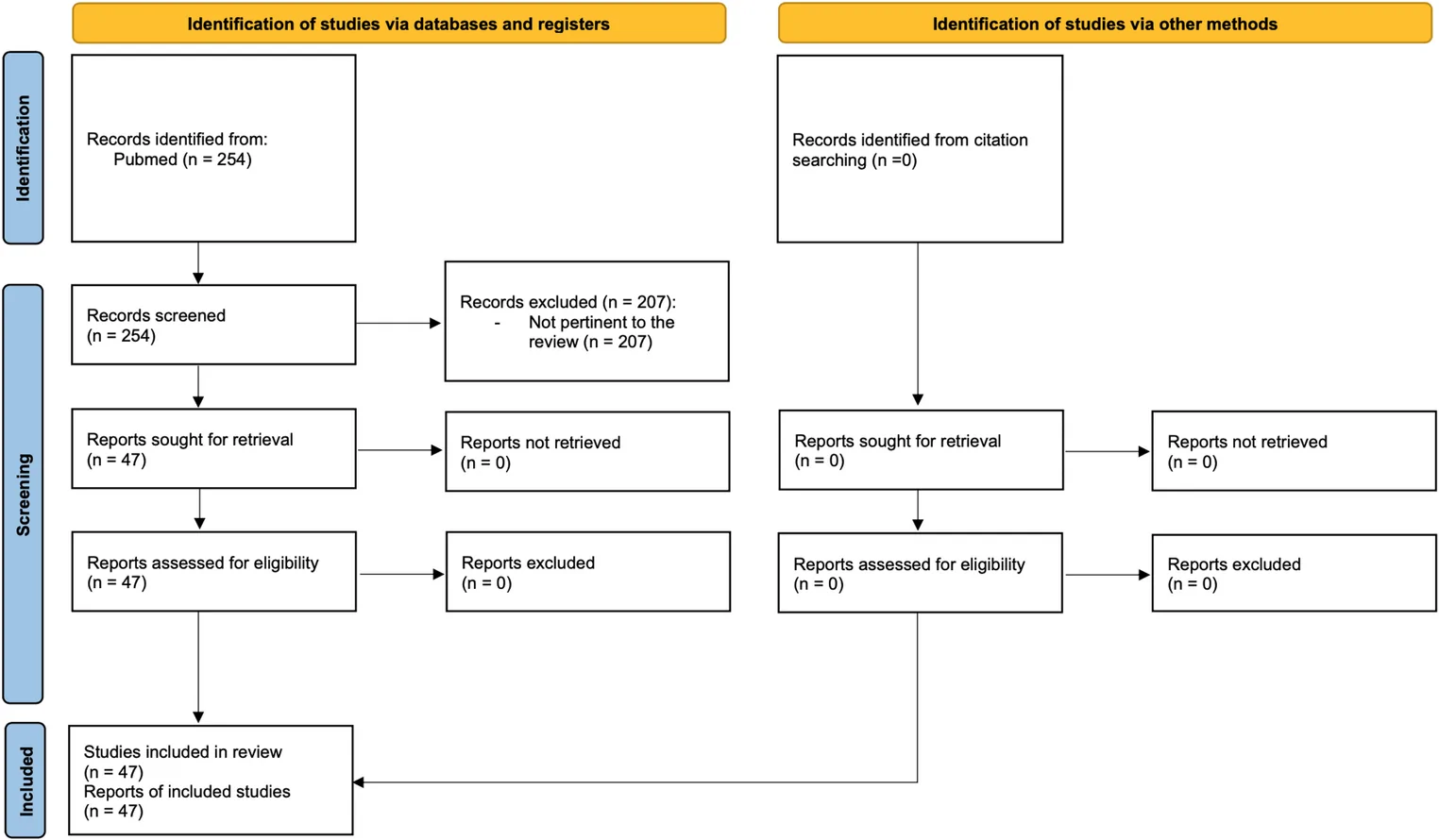
PRISMA flow diagram of study selection

All data were extracted from articles’ text, tables, and figures using the Population, Intervention, Comparison, and Outcome (PICO) framework [13] and included at least the following: title, authors, year of publication, study design, study population, outcomes, and main results. The PRISMA flow diagram (Fig. 1) is provided for transparency of study identification but does not imply a systematic review methodology.

## Automated insulin delivery systems: mechanisms and clinical impact

AID systems integrate three fundamental components: a CGM sensor, an insulin pump and a control algorithm that dynamically modulates insulin delivery in response to real-time glucose data [2, 7]. All currently available AID systems adjust insulin delivery either by modulating preprogrammed basal rates or by delivering micro boluses at frequent intervals (commonly every 5 min), based on CGM feedback, predicted glucose trajectory and rate of glucose change. Different systems predict glucose levels at various time horizons, most commonly 30–60 min ahead, and have either fixed or adjustable glucose targets ranging from 87 to 180 mg/dL (4.8 to 10 mmol/L)^3,7^. All current systems provide automated correction doses, either embedded within the microdose adjustments or as intermittent correction boluses, with frequency and glucose thresholds determined by the specific algorithm [2, 7].

Critically, all current AID systems still require manual entry of carbohydrate content for meal announcements to calculate prandial doses, with the notable exception of the iLet Bionic Pancreas, which, using a fully autonomous and adaptive algorithm, requires only body weight for initialization and a qualitative meal announcement without carbohydrate counting [14].

Three principal classes of control algorithm underpin currently available commercial AID systems [8, 15]: model predictive control (MPC), employed by CamAPS FX, Omnipod 5, DBLG1, and Control-IQ; proportional-integral-derivative (PID), employed by MiniMed 780G; and fuzzy logic (FL), used in the MD-Logic system [8, 16]. Most contemporary HCL systems employ MPC-based algorithms or hybrid architectures that combine predictive modeling with rule-based safety constraints [15].

**Fig. 2:**
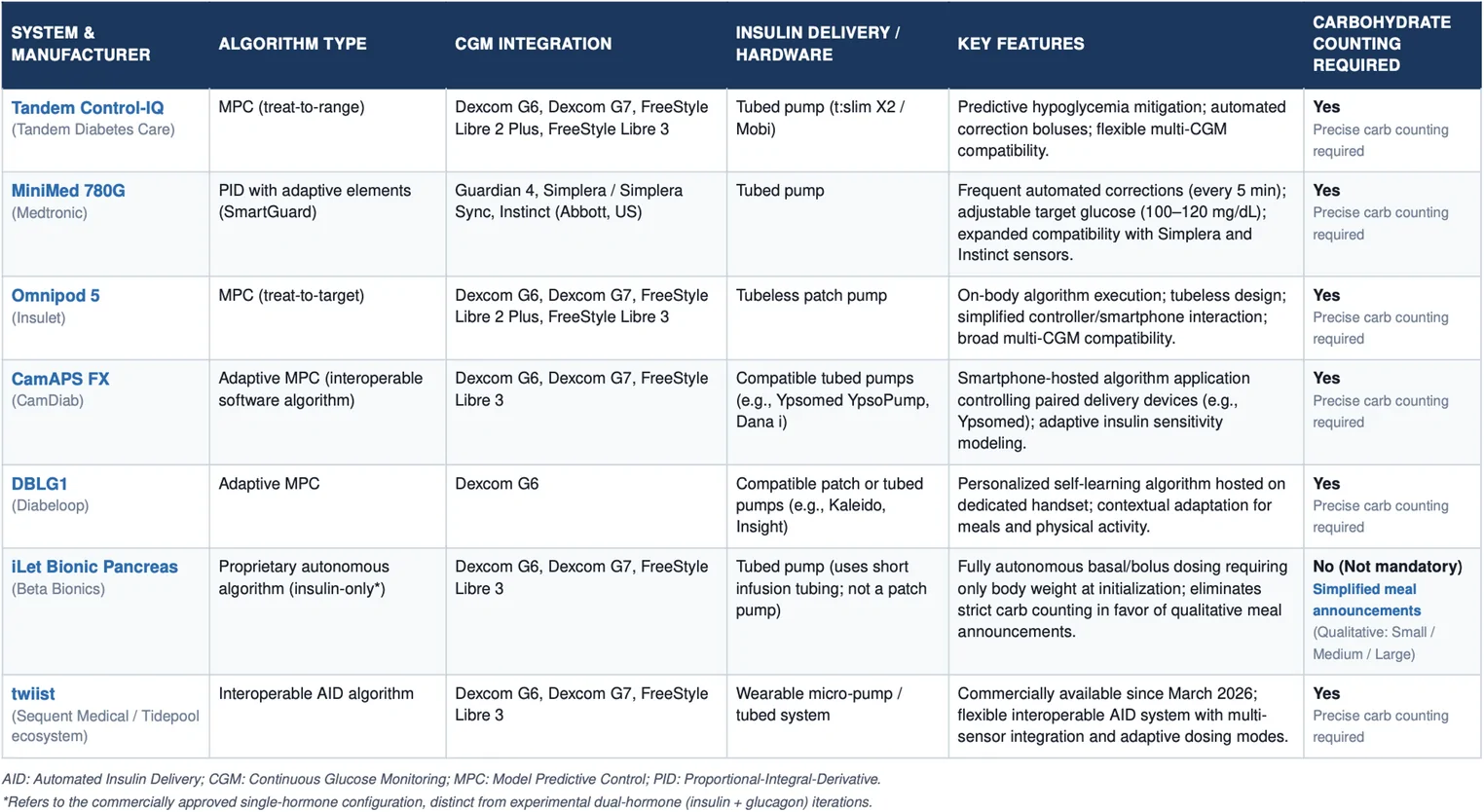
Commercially available AID systems and their key characteristics

Figure 2 summarizes the commercially available AID systems and their key characteristics [2, 7, 14].

### General clinical evidence for aid systems in T1D

Before evaluating evidence specific to older adults, it is essential to establish the foundational glycemic, safety, and psychosocial outcomes demonstrated by AID systems in the general adult T1D population. The following studies represent evidence derived from general T1D adult cohorts (often inclusive of younger participants). These findings illustrate the baseline potential of AID systems prior to age-stratified considerations.

The iDCL trial randomized 168 patients with T1D (age 14–71 years) to closed-loop control (Control-IQ) versus sensor-augmented pump therapy, demonstrating an 11% TIR improvement sustained over 6 months, with significant reductions in both hypoglycemia and HbA1c [17].

Renard et al. specifically evaluated AID in 72 adults at high risk for hypoglycemia, demonstrating a significant reduction in Time Below Range (TBR, < 70 mg/dL) by − 3.7% and an 8.6% increase in TIR [18]. This study provides relevant evidence that AID systems can be effective and safe in individuals at high risk for hypoglycemia, including older adults.

Multiple meta-analyses have consolidated the evidence supporting AID systems across diverse populations. A comprehensive meta-analysis of 12 RCTs demonstrated a mean TIR increase of 10.66%, HbA1c reduction of − 0.34%, and no significant increase in severe hypoglycemia or diabetic ketoacidosis with AID compared with non-automated insulin delivery [19].

A network meta-analysis by Di Molfetta et al. suggested a hierarchy of efficacy among HCL systems: compared with subcutaneous insulin therapy without CGM, MiniMed 780G achieved the highest TIR and the largest reductions in TAR and mean glucose, whereas DBLG1, MiniMed 670G and MiniMed 780G showed the largest reductions in TBR, with no significant differences among them; the risk of severe hypoglycaemia and diabetic ketoacidosis was comparable across insulin therapies [20]. These findings should nevertheless be interpreted with caution. No head-to-head randomised trials comparing commercial AID systems are available, and the reported hierarchy derives from indirect comparisons across trials that differed substantially in comparator therapy, duration, baseline HbA1c, and study population. The resulting between-device rankings should be regarded as hypothesis-generating rather than as evidence of clinical superiority of one system over another — particularly in older adults, in whom no direct comparative data are available.

Moreover, AID systems have demonstrated significant benefits on psychosocial outcomes. A systematic review and meta-analysis by Roos et al. found that AID use was associated with significant reductions in diabetes distress and fear of hypoglycemia [21]. Canha et al. further demonstrated a moderate reduction in caregiver distress associated with AID use [22], a finding of direct relevance to the care of individuals who depend on caregivers for diabetes management. These psychosocial benefits address core barriers to optimal diabetes management and may facilitate sustained technology adoption across diverse populations. Novel report-derived metrics, such as the automated correction index (ACI), have also emerged as promising tools for monitoring system performance and glucose variability in patients on AHCL therapy, potentially informing clinical decision-making and device optimization [23].

## Evidence in older adults

Whereas broad adult T1D trials demonstrate robust TIR improvements and hypoglycemia reductions, older adults represent a distinct cohort requiring dedicated evaluation. Below, we differentiate evidence directly observed in older adults with T1D (aged ≥ 60–65 years) from insights extrapolated from broader adult trials.

Tables 1 and 2 depicts the main characteristics of the studies included in this review. 

**Table 1:** Clinical evidence on AID therapy and diabetes technology in older adults with T1D (RCTs, real-world, longitudinal studies, and systematic reviews)

Study Design	Year	Study population	Mean Age / Age Range	Main Outcomes	TIR & TBR Outcomes	Adherence
**Part A: Studies Specifically Designed for Older Adults**						
Multicenter randomized crossover trial (AIDE-T1D) [11]	2025	* N=82* older adults with T1D	$\:71.4\pm\:4.8\mathrm{\:y}$ ($\:65\mathrm{--}86\mathrm{\:y}$)	AID (Control-IQ) significantly improved glycemic control and reduced hypoglycemia compared to SAP; safe in MCI subgroup	TIR increased by $\:+8.9\mathrm{\:pp}$; TBR70 reduced from 2.57% to 1.58%.	High engagement; AID automated mode use $\:>95\%$.
Prespecified analysis of extension phase (AIDE-T1D) [27]	2025	* N=82* older adults with T1D	$\:71.4\pm\:4.8\mathrm{\:y}$ ($\:65\mathrm{--}86\mathrm{\:y}$)	Glycemic improvements and safety were sustained over 6 months; high treatment satisfaction.	TIR maintained at $\:\sim\:75\%$ over 6-month extension phase.	$\:100\%$ of participants elected to continue HCL therapy.
Randomized crossover trial (ORACL) [25]	2022	* N=30* older adults with T1D	$\:67.1\pm\:5.1\mathrm{\:y}$ ($\:\ge\:60\mathrm{\:y}$)	Closed-loop improved glycemic control vs. SAP, with predominant benefits observed overnight.	TIR $\:+6.2\mathrm{\:pp}$ ($\:75.2\%$ vs. $\:69.0\%$); TBR70 reduced by $\:0.5\mathrm{\:pp}$ (24 h) and $\:0.8\mathrm{\:pp}$ (overnight).	Sensor and pump active wear $\:>90\%$.
Open-label, multicentre, multinational randomized crossover trial [26]	2022	* N=37* older adults with T1D	$\:68.0\pm\:4.5\mathrm{\:y}$ ($\:\ge\:60\mathrm{\:y}$)	CamAPS FX improved TIR vs. SAP; all severe hypoglycemia events occurred during SAP phase	TIR $\:+8.6\mathrm{\:pp}$ ($\:79.9\%$ vs. $\:71.4\%$); significant reduction in TBR	High closed-loop utilization rate.
Prospective pilot study[28]	2022	* N=15* older adults with T1D	$\:72.3\pm\:5.1\mathrm{\:y}$ ($\:\ge\:65\mathrm{\:y}$)	Control-IQ improved TIR, reduced diabetes distress, and improved sleep quality in older adults.	TIR increased by $\:+10\mathrm{\:pp}$ (reaching $\:79.6\%$).	High automated mode use ($\:92.6\%$).
Cross-sectional real-world study (US CareLink database) [12]	2025	$\:N=\mathrm{8,542}$ older adults with T1D/T2D	$\:71.2\pm\:4.9\mathrm{\:y}$ ($\:\ge\:65\mathrm{\:y}$)	MiniMed 780G real-world effectiveness confirmed in older adults, including those $\:\ge\:75\mathrm{\:y}$.	Mean TIR $\:78.4\%$, TBR70 $\:0.9\%$; with recommended settings TIR $\:81.9\%$, TITR $\:55.9\%$.	High real-world engagement and feature utilization.
Retrospective real-world study[29]	2022	* N=48* older adults with T1D	$\:70.1\pm\:4.2\mathrm{\:y}$ ($\:65\mathrm{--}81\mathrm{\:y}$)	Closed-loop therapy yielded immediate and marked glycemic improvements in older adults.	TIR increased from $\:62\%$ to $\:76\%$ ($\:+14\mathrm{\:pp}$); marked TBR reduction.	High system retention ($\:>90\%$).
Prospective observational real-world study (1-year follow-up) [30]	2023	* N=42* older adults with T1D	$\:74.1\pm\:5.6\mathrm{\:y}$ ($\:\ge\:65\mathrm{\:y}$)	AHCL (MiniMed 780G) provided sustained glycemic improvements and safety over 12 months.	TIR increased from $\:64\%$ to $\:79.8\%$; TBR70 stabilized at $\:1.2\%$.	$\:100\%$ treatment continuity over 1 year.
Cross-sectional analysis of cohort study (SOLID) [42]	2020	* N=801* older adults with T1D	$\:69.2\pm\:4.7\mathrm{\:y}$ ($\:\ge\:60\mathrm{\:y}$)	Severe hypoglycemia strongly associated with impaired global cognition in older T1D.	High TBR/severe hypoglycemic events tripled cognitive impairment risk.	N/A (observational cohort).
Systematic review and meta-analysis of RCTs[10]	2025	5 RCTs (* N=482* older adults with T1D)	$\:68.5\mathrm{\:y}$ (pooled mean age, $\:\ge\:60\mathrm{\:y}$)	First meta-analysis dedicated to older adults; confirmed high efficacy and drastic hypo risk reduction.	TIR $\:+7.90\%$ ($\:95\%\mathrm{\:CI\:}7.09\mathrm{--}8.72$); $\:84\%$ reduction in severe hypo risk ($\:\mathrm{Peto\:OR\:}0.16$).	High overall intervention retention across RCTs.

Study Design	Year	Total population & Older Subgroup *N*	Age Range & Overall Mean Age	Older Adult Subgroup Analysis Performed?	Key Outcomes & Glycemic Impact (TIR/TBR)	Adherence / Tech Metrics
Part B: Studies Including Older Adults Within Broader Adult Populations						
Multicenter RCT [14]	2022	* N=219* T1D adults (* N=38* aged $\:\ge\:65\mathrm{\:y}$)	$\:18\mathrm{--}78\mathrm{\:y}$ ($\:41\pm\:13\mathrm{\:y}$)	**Yes** (Prespecified subgroup analysis by age, including ≥ 65 y)	iLet Bionic Pancreas improved HbA1c ($\:0.5\mathrm{\:pp}$) and TIR ($\:+11\mathrm{\:pp}$); glycemic benefits consistent in older adults.	High retention; reduced cognitive burden.
Randomized crossover trial [48]	2025	* N=34* T1D adults (Includes older spectrum)	$\:18\mathrm{--}72\mathrm{\:y}$ ($\:46.8\pm\:14.2\mathrm{\:y}$)	**No** (Overall cohort comparison of meal strategies)	Simplified meal announcements achieved TIR $\:80.5\%$ vs. $\:77.7\%$ (precise); reduced level 1 TBR ($\:1.9\%$ vs. $\:2.8\%$).	Reduced daily cognitive/meal management burden.
Retrospective real-world study (international CareLink database) [31]	2025	$\:N=\mathrm{14,211}$ users ($\:\ge\:56\mathrm{\:y}$ focus cohort)	$\:\ge\:56\mathrm{\:y}$ ($\:63.8\pm\:6.1\mathrm{\:y}$)	**Yes** (Stratified analysis by age brackets)	MiniMed 780G users $\:\ge\:56\mathrm{\:y}$ achieved TIR $\:77.1\%$ ( $\:>80\%$ with recommended settings); performed equal/superior to younger cohorts.	High overall pump/CGM wear time.
Multinational real-world evidence analysis [32]	2023	$\:N=\mathrm{1,813}$ CamAPS FX users (* N=124* aged $\:\ge\:65\mathrm{\:y}$)	1–81 y	**Yes** (Age-stratified real-world analysis)	TIR increased monotonically with age, reaching the highest value in the $\:\ge\:65\mathrm{\:y}$ cohort ($\:81.8\%$).	Closed-loop wear time highest in older adults ($\:94.7\%$)
Prospective longitudinal cohort study (DCCT/EDIC) [41]	2021	$\:N=\mathrm{1,051}$ T1D adults followed over 32 years	$\:59.2\pm\:6.8\mathrm{\:y}$ at 32y evaluation	**Yes** (Longitudinal aging cohort evaluation)	Severe hypoglycemia linked to psychomotor decline equivalent to $\:4.6\mathrm{\:y}$ of additional cognitive aging.	N/A (observational cohort).
Retrospective cohort study[43]	2021	$\:N=\mathrm{2,821}$ T1D adults $\:\ge\:50\mathrm{\:y}$	$\:\ge\:50\mathrm{\:y}$ ($\:64.5\pm\:8.9\mathrm{\:y}$)	**Yes** (Stratified analysis by age and event frequency)	Severe hypoglycemia and hyperglycemia independently elevated long-term clinical dementia risk by $\:66\%$.	N/A (observational cohort).
Systematic review and meta-analysis [8]	2018	40 studies ($\:N=\mathrm{1,027}$ T1D patients across age groups)	Broad age range across included trials	**No** (General T1D population meta-analysis)	Outpatient AID overall improved TIR by $\:+9.62\%$ and reduced hypoglycemia compared to non-AID.	High user satisfaction across studies.
Systematic review and meta-analysis [19]	2026	12 RCTs ($\:N=\mathrm{1,140}$ T1D participants)	Broad age range across included trials	**Yes** (Subgroup analysis by population characteristics)	AID improved TIR by $\:+10.66\%$ and reduced HbA1c consistently across diverse population subgroups.	High safety profile supported continuous wear.
Systematic review and network meta-analysis [20]	2024	28 RCTs ($\:N=\mathrm{2,215}$ T1D participants)	Broad age range across included trials	**No** (Network comparison of AID systems overall)	MiniMed 780G and DBLG1 ranked highest for TIR optimization and TBR reduction overall.	High usability across HCL platforms.
Systematic review and meta-analysis [21]	2024	62 studies ($\:N=\mathrm{9,253}$ participants)	Broad age range across included studies	**Yes** (Subgroup analysis for adult populations)	AID therapy significantly reduced diabetes distress ($\:\mathrm{SMD\:}-0.159$) and fear of hypo ($\:\mathrm{SMD\:}-0.339$).	Improved compliance driven by lower distress.
Systematic review and meta-analysis [22]	2024	18 studies ($\:N=\mathrm{2,410}$ caregivers/patients)	Broad age range across included studies	**Yes** (Subgroup analysis for caregiver/dependent adults)	AID significantly reduced caregiver distress ($\:\mathrm{SMD\:}-0.48$ in observational studies).	Enhanced caregiver-assisted adherence.
Systematic review and meta-analysis [50]	2025	4 RCTs (* N=137* T1D patients)	Broad age range across included trials	**No** (Overall meta-analysis on simplified meal algorithms)	Simplified meal announcements resulted in minimal TIR difference ($\:-3.28\mathrm{\:pp}$) vs. exact carb counting	Lower user burden favoring long-term adherence.

**Table 2:** Clinical practice guidelines, consensus statements, narrative reviews and qualitative/usability studies

Type of Document	Year	Focus Population / Subject	Key Summary / Recommendations
Clinical practice guideline [2]	2026	Older Adults with Diabetes	Recommends individualized glycemic goals (e.g., TIR $\:>50\%$, TBR $\:<1\%$ for frail patients); emphasizes screening for cognitive impairment and hypoglycemia risk.
Consensus report (ADA/EASD) [4]	2021	Adults with Type 1 Diabetes	Recommends structured education, technology adoption (CGM/AID), and individualized targets aligned with functional status and life expectancy
Consensus report (EASD/ADA) [7]	2022	AID Systems in Diabetes	Provides comprehensive consensus on AID benefits, challenges, infusion site management, and strategies to prevent alarm fatigue in older adults.
Clinical practice guideline [24]	2026	Diabetes Technology	Endorses early AID initiation in T1D across the lifespan; highlights CGM integration, device accessibility, and caregiver support strategies.
Clinical practice guideline (Endocrine Society) [40]	2023	High Hypoglycemia Risk Populations	Recommends real-time CGM and AID technologies as first-line tools to prevent severe hypoglycemia in vulnerable and older patients.
Clinical practice guideline [45]	2026	Glycemic Goals & Hypoglycemia	Establishes standard clinical targets (TIR $\:>70\%$, TBR $\:<4\%$) and relaxed targets (TIR $\:>50\%$, TBR $\:<1\%$) for older or complex adults.
Clinical practice guideline (Endocrine Society) [46]	2019	Older Adults with Diabetes	Advocates for stratifying older adults by health/functional status (Healthy, Complex, Very Complex) to guide HbA1c and tech choices.
Narrative review [1]	2014	Older Adults ($\:\ge\:65\mathrm{\:y}$) with T1D	Early overview of T1D management in aging populations; highlights heightened hypoglycemia vulnerability and geriatric syndromes.
Expert review / Scientific statement [3]	2025	Older Adults ($\:\ge\:65\mathrm{\:y}$) with T1D	Identifies care challenges including misdiagnosis, cognitive decline, physical disability, and the essential role of caregiver involvement.
Narrative review [5]	2017	Older Adults with Diabetes	Details frailty and sarcopenia as emerging complications that complicate diabetes self-management and technology utilization.
Narrative review [15]	2021	Closed-Loop Systems	Evaluates technical aspects of closed-loop algorithms (MPC, PID) and the importance of simplified interfaces for older users.
State-of-the-art review (JACC) [35]	2020	Gericardiology / Gerotechnology	Discusses digital health and device usability in older adults with cardiovascular co-morbidities and sensory impairments.
Scoping review [36]	2026	Older Adults ($\:\ge\:65\mathrm{\:y}$)	Analyzes barriers to digital health adoption using Innovation Resistance Theory (functional, usability, and psychological barriers).
Narrative review [37]	2024	Sensory-Impaired Populations	Examines disparities in diabetes tech access for deaf/hard-of-hearing and blind/low-vision individuals, calling for accessible AID design.
Expert review [38]	2025	Older Adults with Diabetes	Focuses on clinical implementation strategies, patient selection, tailored training, and caregiver integration for AID success.
Expert review / Roadmap [39]	2020	Aging Population with Diabetes	Calls for age-friendly technologies, simplified device interfaces, and dedicated clinical trial evidence for older adults.
Expert review / Scientific statement [47]	2023	Older Adults with Diabetes	Outlines the expanding role of CGM, AID, and remote monitoring in geriatric care; addresses practical implementation hurdles.
Narrative review [49]	2024	Aging Well with Diabetes	Explores how AID-mediated hypoglycemia prevention attenuates frailty progression and improves quality of life in older adults.
Companion analysis of RCT (PROs) [33]	2023	Older Adults ($\:\ge\:60\mathrm{\:y}$) with T1D	CamAPS FX use significantly improved diabetes distress and powerlessness; high preference for HCL over SAP therapy
Qualitative study (nested within ORACL RCT) [34]	2023	Older Adults ($\:\ge\:60\mathrm{\:y}$) with T1D	Qualitative analysis showing AID reduced cognitive self-care burden and improved sleep, despite minor alarm-management challenges.
Cross-sectional qualitative study [51]	2025	Older Adults $\:\ge\:75\mathrm{\:y}$ with Diabetes	Evaluates device usability in adults $\:\ge\:75\mathrm{\:y}$; highlights preferences for larger screen fonts, simple menus, and long battery life.

### Randomized controlled trials

Evidence regarding the use of AID systems in older adults with T1D has historically been limited, largely because individuals aged ≥ 65 years have been systematically underrepresented in diabetes technology trials. Nevertheless, recent studies specifically targeting this population have begun to address this important knowledge gap [2, 24].

The first randomized controlled trial specifically designed for older adults with T1D was the Older Adults Closed Loop (ORACL) study [25]. In this open-label, multicentre, crossover trial, 30 participants aged ≥ 60 years with long-standing T1D were randomized to receive four months of closed-loop therapy and four months of sensor-augmented pump (SAP) therapy. Closed-loop treatment significantly improved glycaemic control, increasing TIR from 69.0% during SAP therapy to 75.2%. All prespecified CGM metrics favoured closed-loop therapy, with the most pronounced benefits observed overnight. TBR was reduced by 0.5%over 24 h and by 0.8%during the night. Although no significant difference in HbA1c was observed, the study demonstrated that older adults could safely and effectively use closed-loop technology, achieving improved glucose control while minimizing hypoglycaemia exposure [25].

Additional evidence was provided by a multicentre, multinational, randomized crossover trial using the CamAPS FX hybrid closed-loop system. 37 adults aged ≥ 60 years underwent two 16-week treatment periods comparing HCL and SAP therapy. HCL significantly increased TIR compared with SAP therapy (79.9% vs. 71.4%), without increasing hypoglycaemia risk. All severe hypoglycaemia episodes occurred during the SAP period, further supporting the safety profile of automated insulin delivery in this population [26].

The largest trial specifically targeting older adults was the multicentre randomized crossover AIDE-T1D study. 82 participants aged between 65 and 86 years underwent sequential 12-week periods of HCL, PLGS, and SAP therapy. TBR was significantly reduced with both HCL and PLGS compared with SAP therapy, decreasing from 2.57% during SAP use to 1.58% and 1.67% during HCL and PLGS therapy, respectively. Importantly, HCL therapy also resulted in superior TIR, reduced hyperglycaemia, and lower HbA1c values compared with both comparator strategies. Automated mode use exceeded 95% of the study duration, highlighting the feasibility of AID even among individuals in their eighth and ninth decades of life [11].

Longer-term data from the extension phase of AIDE-T1D further strengthened these findings. Among participants completing the randomized phase, 91% elected to continue HCL therapy, while only a minority selected PLGS or non-automated approaches. Glycaemic improvements were maintained during follow-up, with mean TIR remaining approximately 75%, and no new safety concerns emerged [27].

Finally, a small prospective pilot study evaluating the Control-IQ system in adults aged ≥ 65 years demonstrated not only improvements in glycaemic outcomes but also significant reductions in diabetes-related distress and high levels of satisfaction, trust, and perceived usability. Although limited by its small sample size, this study highlighted potential psychosocial benefits of AID systems that may be particularly relevant in older populations [28].

Overall, the currently available randomized evidence consistently demonstrates that AID systems improve glycaemic control while reducing hypoglycaemia exposure in older adults with T1D. Across studies, improvements in TIR ranged from approximately 6% to 9%, accompanied by reductions in TBR and high levels of device adherence. Importantly, these benefits were achieved without increasing adverse events, suggesting that older adults can successfully adopt and maintain advanced diabetes technologies despite the greater prevalence of comorbidities, functional limitations, and age-related physiological changes.

Table 3 provides a structured overview of the principal benefits and limitations of AID systems in older adults with T1D, as emerging from the available randomized and real-world evidence.

**Table 3:** Benefits and limitations of closed-loop insulin delivery systems in older adults with type 1 diabetes

PROs	CONs	Ref.
**Glucose control**		
TIR improvement of ~ 6–10 pp vs. SAP, confirmed across RCTs and real-world studies	HbA1c improvement modest and not always statistically significant in trials (baseline already well-controlled ~ 7.4%)	[2, 8, 10, 11, 25, 26]
Significant reduction in time below range (TBR 3.9 and 3.0 mmol/L), especially overnight	TBR benefit is not uniform across studies (significant in ORACL and AIDE-T1D, not significant in Boughton et al.)	[11, 25, 26]
Particularly marked overnight benefits (overnight TIR + 14.4 pp in Boughton et al.)	Isolated episodes of severe hypoglycemia still reported during CL use (3 events in ORACL)	[25, 26]
**Safety**		
No increase in serious adverse events compared with SAP in dedicated trials	Risk of DKA in case of pump malfunction or infusion interruption	[7, 15, 25, 26, 48]
Reduction in hypoglycemia exposure particularly relevant in patients with impaired awareness of hypoglycemia (IAH)	Pharmacological interferences (e.g., acetaminophen) may cause falsely elevated CGM readings	[7, 27, 47, 50]
Safety profile comparable to that observed in younger populations	CGM not adequately tested in patients with GFR 30 mL/min, a condition prevalent among older adults	[11, 25, 33, 46, 50]
**Psychosocial Benefits and Quality of Life**		
Reduction in diabetes-related distress and fear of hypoglycemia	Gap between expectations and reality of CL therapy: some participants reported disappointment	[21, 22, 29, 41, 43]
Improved sleep quality and reduced overnight anxiety	Alarm fatigue: nocturnal alarms perceived as disruptive and a source of frustration	[29, 41, 43]
**Usability and Age-Related Barriers**		
Automated mode adherence consistently > 90% of time across trials	Visual and hearing deficits may impair display reading and alarm perception	[11, 25, 26, 40, 45, 48]
Older adults with regular daily routines may achieve glycemic outcomes equal to or exceeding those of younger users	Low digital literacy and limited smartphone familiarity may restrict app integration	[12, 24, 31, 33, 34, 46]
Remote caregiver monitoring already technically feasible	Nursing home and long-term care staff often lack training in pump and CGM management	[2, 3, 27, 34, 45, 46]

Abbreviations: TIR = Time in Range; TBR = Time Below Range; TBR3.9 = Time Below 3.9 mmol/L (70 mg/dL); TBR3.0 = Time Below 3.0 mmol/L (54 mg/dL); SAP = Sensor-Augmented Pump; RCTs = Randomized Controlled Trials; HbA1c = Glycated Hemoglobin; CL = Closed Loop; DKA = Diabetic Ketoacidosis; IAH = Impaired Awareness of Hypoglycemia; CGM = Continuous Glucose Monitoring; GFR = Glomerular Filtration Rate; PROs = Patient-Reported Outcomes; CKD = Chronic Kidney Disease; ADA = American Diabetes Association; EASD = European Association for the Study of Diabetes; ORACL = Older Adult Closed Loop Study; AIDE-T1D = Automated Insulin Delivery in Elderly with Type 1 Diabetes

### Real-world evidence

Real-world studies are particularly important in older adults, whose clinical complexity and heterogeneity are often insufficiently represented in trial populations.

At the population level, the effectiveness of AID in routine care has been confirmed by large national registry analyses. In an analysis of the Swedish National Diabetes Register including adults with T1D followed between 2014 and 2024, AID use was associated with significantly lower HbA1c than multiple daily injections combined with CGM (β = −4.0 mmol/mol; 95% CI − 4.3 to − 3.7; *P* < 0.001) after adjustment for age, diabetes duration, sex, body mass index, physical activity, smoking, year, and centre size, with consistent benefits across systems (− 4.8 mmol/mol for Tandem Control-IQ, − 3.1 mmol/mol for MiniMed 780G, and − 2.9 mmol/mol for MiniMed 670G), whereas conventional pump therapy with CGM conferred a more modest advantage (− 1.7 mmol/mol). This analysis, however, included adults aged ≥ 18 years, with age entered as an adjustment covariate rather than as an effect modifier and no subgroup analysis reported for individuals aged ≥ 65 years; moreover, the outcome assessed was HbA1c rather than CGM-derived metrics, which are generally preferred in older adults given the limited reliability of HbA1c and the priority of hypoglycaemia exposure as a safety outcome in this population. Studies specifically addressing older adults are therefore required to determine whether these population-level benefits are reproduced at advanced age [29].

The first real-world study specifically focused on older adults with T1D was reported by Toschi and colleagues. In this retrospective analysis of 48 patients with a mean age of 70 years and a mean diabetes duration exceeding four decades, initiation of HCL therapy resulted in substantial improvements in glycaemic control. After three months, TIR increased from 62% to 76%, accompanied by reductions in both hypoglycaemia and hyperglycaemia exposure. Importantly, system uptake was high, suggesting that older adults with extensive diabetes experience can successfully adopt advanced diabetes technologies in everyday practice [30].

Longer-term prospective evidence was subsequently provided by Pintaudi et al., who evaluated the MiniMed 780G AHCL system in older adults with T1D over a one-year period. In this cohort, with a mean age of 74 years, TIR increased from approximately 64% at baseline to nearly 80% after 12 months of follow-up. These improvements were maintained throughout the study period, supporting the durability of AID benefits over time. TBR remained consistently low, confirming the favourable safety profile of the system [31].

More recently, large-scale analyses of cloud-based platform data have provided valuable information regarding AID performance in thousands of older users. An analysis of CareLink data from 8,542 MiniMed 780G users aged ≥ 65 years demonstrated excellent glycaemic outcomes, with mean TIR exceeding 78% and TBR remaining below 1%. Among users adopting recommended system settings, TIR exceeded 80%, while glycaemic metrics remained similarly favourable in individuals aged ≥ 75 years. These findings suggest that advanced age does not limit the effectiveness of automated insulin delivery, and that optimal system configuration may further enhance outcomes [12].

Similarly, a large real-world analysis comparing older and younger users of the MiniMed 780G system found that older mean TIR exceeded 77% in older users and increased beyond 80% among those utilizing recommended settings, while hypoglycaemia exposure remained very low. These observations challenge the traditional perception that older age may represent a barrier to successful technology use [32].

Comparable findings have been reported with other AID platforms. A multinational analysis of more than 1,800 CamAPS FX users across 15 countries demonstrated that glycaemic outcomes improved progressively with age, with users aged ≥ 65 years achieving the highest TIR of all age groups [33].

Taken together, real-world studies consistently confirm the efficacy and safety of AID systems in older adults. Improvements in TIR observed in clinical practice are remarkably similar to those reported in randomized trials and are accompanied by very low rates of hypoglycaemia. Interestingly, several studies suggest that older adults may derive benefits that are comparable to, or even greater than, those observed in younger individuals. Potential explanations include higher treatment adherence, more consistent daily routines, and greater engagement with diabetes self-management.

### Usability and acceptance

The successful implementation of AID systems in older adults extends beyond glycaemic efficacy and requires careful consideration of usability, treatment burden, psychosocial impact, and the individual’s cognitive and functional capacity. While chronological age alone should not be considered a barrier to technology adoption, age-related changes in cognition, vision, hearing, manual dexterity, and digital literacy may influence the ability to safely and effectively use advanced diabetes technologies.

The American Diabetes Association (ADA) recommends that eligibility for AID systems should be based on the individual’s ability to operate the technology safely and consistently, considering cognitive function, functional status, health literacy, and the availability of caregiver support when appropriate. AID systems are now considered the standard of care for people with T1D who are capable of using them safely and effectively [2].

Despite concerns regarding technological complexity, adherence to AID systems has been remarkably high across studies involving older adults. In the Control-IQ pilot study conducted by Bisio et al.^10^ participants used the automated mode for 92.6% of available time and reported high levels of trust, ease of use, and overall usability. Similarly, the extension phase of the AIDE-T1D trial demonstrated sustained engagement, with 91% of participants selecting hybrid closed-loop therapy as their preferred treatment modality following completion of the randomized phase [27].

Beyond traditional glycaemic outcomes, recent studies have also explored psychosocial and patient-reported outcomes. A companion analysis of the CamAPS FX trial evaluated patient-reported outcomes in 35 participants aged ≥ 60 years. After 16 weeks of hybrid closed-loop use, significant improvements were observed in overall diabetes distress, particularly in the domains of powerlessness and physician-related distress. Participants reported greater confidence in glucose management, improved trust in CGM data, and reduced emotional burden associated with daily diabetes self-care. Qualitative interviews consistently identified improved glycaemic control and reduced worry about diabetes as the most important perceived benefits of closed-loop therapy [34].

Qualitative investigations have provided additional insights into the lived experience of older adults using AID systems. Semi-structured interviews conducted among participants in the ORACL trial identified several recurring themes, including improved sleep quality, reduced diabetes-related psychological burden, increased confidence in glucose management, and enhanced quality of life. Many participants described feeling liberated from the constant vigilance traditionally associated with diabetes self-management. However, these studies also highlighted a gap between expectations and real-world experience in some individuals, emphasizing that technological benefits are not universally perceived and may be influenced by personal preferences, prior technology experience, and treatment expectations [35].

Several barriers may limit the adoption and sustained use of AID systems in older adults. Age-related cognitive and physical challenges represent important obstacles to successful technology utilization. Cognitive decline, executive dysfunction, reduced manual dexterity, visual impairment, and hearing loss may all interfere with the routine operation of diabetes technologies, including continuous glucose monitoring and automated insulin delivery systems. As highlighted by Krishnaswami and colleagues, the cumulative burden of cognitive, sensory, and functional impairments may complicate technology adoption and reduce the ability of some older adults to independently manage increasingly sophisticated diabetes devices [36].

Nutritional and behavioral factors may represent additional challenges in frail older adults. Irregular meal patterns, reduced appetite, malnutrition, gastroparesis, and inaccurate meal announcements may increase glycemic variability and reduce the effectiveness of current AID algorithms, which still largely depend on user-initiated meal input. In this context, simplified meal announcement strategies and fully adaptive systems may be particularly relevant for reducing treatment burden in vulnerable older individuals.

Applying the Innovation Resistance Theory framework, Birati and Tzemah-Shahar [37] identified multiple determinants of resistance to digital health technologies among older adults. Functional barriers included interface complexity, usability challenges, and age-related impairments that hinder interaction with digital devices. Psychological resistance was driven by emotional discomfort, technology-related anxiety, and concerns regarding the potential loss of personal relationships with healthcare providers. In addition, value- and risk-related concerns, including distrust in device accuracy, privacy issues, and data security, were frequently reported. Together, these factors may contribute to reduced self-efficacy and reinforce avoidance behaviours toward digital health solutions [37].

Additional disparities in technology access and utilization have been documented among individuals with visual impairment, blindness, deafness, or hearing loss. These populations frequently encounter structural barriers that limit access to diabetes technologies, highlighting the need for more inclusive device design and greater attention to accessibility when developing future AID platforms [38].

Given these challenges, successful implementation of AID systems in older adults requires a personalized and geriatric-focused approach. Urbina and colleagues emphasized that careful patient selection, comprehensive education, and active caregiver involvement are critical determinants of successful technology adoption [39].

Finally, Munshi and colleagues have advocated for the development of age-friendly diabetes technologies specifically tailored to the needs of older adults. Proposed strategies include incorporating cognitive support tools such as reminders, alerts, and caregiver-interaction modules, as well as improving device usability through larger buttons, simplified interfaces, enhanced screen contrast, and more intuitive workflows. Such adaptations may help extend the benefits of AID systems to a broader and more clinically diverse population of older adults living with T1D [40].

## Safety considerations

Severe hypoglycemia is associated with serious adverse outcomes, including falls, fractures, cardiac arrhythmias, cardiovascular events, delirium, functional decline, loss of independence, hospitalizations, cognitive decline, and increased mortality. Aging is accompanied by physiological alterations that further increase susceptibility to hypoglycemia, such as impaired counterregulatory hormonal responses, reduced symptom perception, diminished renal function, and altered insulin pharmacodynamics [1]. Moreover, recurrent hypoglycemia may contribute to impaired awareness of hypoglycemia (IAH) and neurocognitive dysfunction, creating a bidirectional vicious cycle: cognitive decline increases the risk of hypoglycemia, and severe hypoglycemia accelerates further cognitive deterioration [2, 41].

In the DCCT/EDIC cohort (1,051 adults with T1D followed for 32 years), one or more episodes of severe hypoglycemia were independently associated with a decline in psychomotor function and mental efficiency equivalent to 4.6 additional years of age, with a dose-response relationship observed with increasing number of events [42]. In the Study of Longevity in Diabetes (SOLID), recent severe hypoglycemia in older adults with T1D was associated with a threefold increased risk of impaired global cognition [43]. Furthermore, Whitmer et al. demonstrated that in 2,821 older adults with T1D, exposure to severe hypoglycemic events was associated with a 66% increased risk of developing dementia [44]. These data underscore the particular vulnerability of older adults with T1D to hypoglycemia-related complications and highlight the importance of comorbid conditions, including those frequently encountered in geriatric settings, such as acute illness and metabolic derangements, that may further compound glycemic instability [45].

For these reasons, international guidelines recommend individualized glycemic targets in older adults, prioritizing hypoglycemia prevention and reduction of treatment burden, especially in frail patients or those with limited life expectancy [2]. The ADA Standards of Care in Diabetes 2026 recommend that for older adults aged ≥ 65 years with intermediate or complex health, the CGM target for TBR be limited to less than 1% (compared with ≤ 4% in healthy older adults) and TIR exceed 50% (compared with ≥ 70%), with further relaxation of targets in individuals with very complex or poor health status [2, 46]. The ADA also recommends considering the use of AID systems to reduce hypoglycemia risk in older adults, based on individual ability and support system [2].

Across the AIDE T1D, ORACL, and CamAPS FX trials, HCL therapy reduced TBR compared with SAP therapy, with the most pronounced benefits observed during the overnight period [11, 25, 26].

The meta-analysis by Lei et al. (5 RCTs, 482 participants) quantified an 84% reduction in severe hypoglycemia risk [10]. Real-world data further corroborate these findings: in the largest dataset to date, Chien et al. reported a mean TBR of 0.9% among 8,542 MiniMed 780G users aged ≥ 65 years, meeting the stringent international consensus target of < 1%^12^. Taken together, the available evidence supports the role of AID systems as an effective strategy for hypoglycemia risk reduction in older adults, addressing one of the most critical safety concerns in this population.

Rates of diabetic ketoacidosis (DKA) in AID users appear low in older adult populations and comparable to those observed with conventional pump therapy [10, 25]. However, it is important to recognize that DKA risk with pump therapy is primarily related to infusion set failures, which remain the most common cause of hyperglycemia and ketosis events in all AID users [7].

Older adults may be particularly vulnerable to delayed recognition of infusion set occlusion or pump malfunction, especially those with cognitive impairment or visual limitations [36, 40].

In post-acute and long-term care (PALTC) settings, DKA may be mistaken for sepsis, end-organ failure, or other electrolyte abnormalities, underscoring the need for staff education [2].

It is critical to acknowledge that the available evidence is largely derived from relatively robust and cognitively intact older adults. Most RCTs excluded individuals with moderate-to-severe cognitive impairment, advanced frailty, or residence in PALTC facilities [10]. A notable exception is the AIDE T1D trial, in which a prespecified subgroup analysis demonstrated that participants with mild cognitive impairment achieved the same glycemic efficacy as those without cognitive impairment [11].

Future studies specifically designed to include frail older adults, those with moderate-to-severe cognitive decline, and individuals in institutional care settings are urgently needed to define the safety boundaries and optimal implementation strategies for AID in these vulnerable populations [40].

## Clinical implications and future directions

**Fig. 3:**
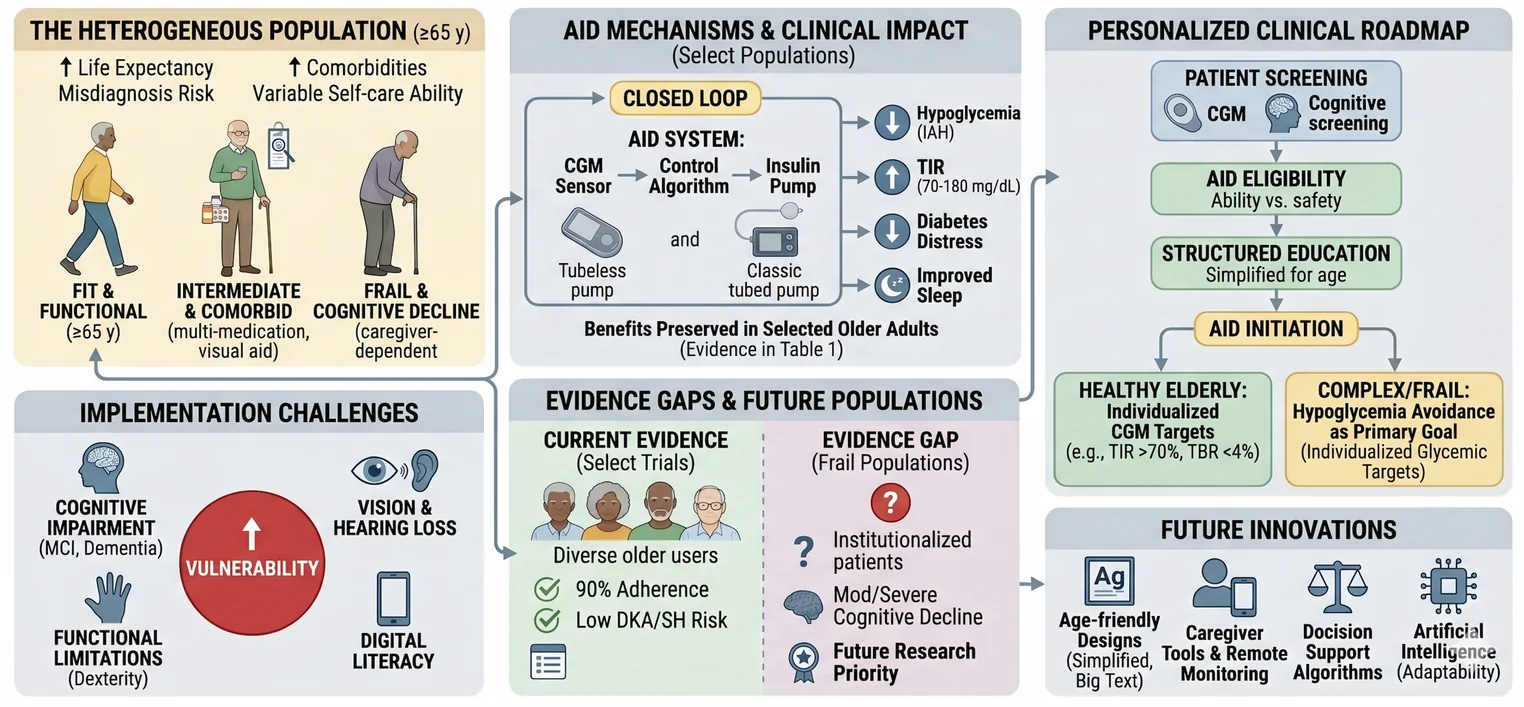
Visual summary of the use of AID systems for older adults with T1D

The expanding evidence base supporting the use of AID systems in older adults with T1D has important implications for clinical practice. Collectively, randomized trials, real-world studies, and patient-reported outcome analyses indicate that the benefits of AID systems extend beyond improvements in glycaemic metrics and include meaningful reductions in hypoglycaemia exposure, treatment burden, and diabetes-related distress. These outcomes are particularly relevant in older adults, for whom the prevention of hypoglycaemia and preservation of functional independence often represent higher priorities than strict glycaemic normalization [2]. Figure 3 visually summarizes the use of AID systems for Older Adults with T1D.

Current evidence supports the use of AID systems in older adults who are willing and able to engage with diabetes technology, particularly those with impaired awareness of hypoglycaemia, recurrent severe hypoglycaemic events or marked glycaemic variability [2, 41, 47]. Older adults with long-standing T1D may derive substantial benefit from the automated adjustment of insulin delivery, especially during the overnight period, when hypoglycaemia frequently occurs and often remains unrecognized [2, 25, 41, 47]. Importantly, eligibility for AID therapy should not be determined by chronological age alone. Rather, a comprehensive geriatric assessment should include cognitive function, functional status, visual and hearing abilities, manual dexterity, health literacy, and familiarity with digital technologies. Equally important is the evaluation of social circumstances and the availability of caregiver support [2, 24]. As the population of older adults with T1D continues to increase, age-related cognitive decline, frailty, and functional impairments are likely to become increasingly common [2]. In this context, caregivers may play a pivotal role in facilitating the safe and effective use of AID systems, maintaining the benefits of advanced diabetes technology while reducing the risk of treatment-related complications [7, 48, 49]. Future clinical models should increasingly incorporate caregivers into technology education, device management, and remote monitoring strategies.

Importantly, older adults with T1D should not be considered a homogeneous population. Clinical phenotypes may range from healthy and independent individuals to frail patients with cognitive impairment, disability, or residence in long-term care facilities (Table 4). In frail older adults, the value of AID systems may extend beyond glycemic optimization and include preservation of functional autonomy, reduction of caregiver burden, prevention of hypoglycemia-related falls, and simplification of diabetes self-management. In this context, AID systems may increasingly be viewed not only as glucose-lowering technologies, but also as assistive technologies supporting functional and cognitive compensation.

**Table 4:** Clinical phenotypes and practical considerations for AID therapy in older adults with T1D

Clinical phenotype	Potential benefits of AID	Main limitations/risks	Practical considerations
Healthy/independent older adult	Improved TIR, reduced hypoglycemia, lower treatment burden, improved quality of life	Alarm fatigue, technological burden	Standard AID implementation, individualized glycemic targets, structured education
Frail older adult	Prevention of hypoglycemia, reduction of glycemic variability, simplified insulin management, preservation of functional autonomy	Nutritional variability, sarcopenia, functional dependence, increased vulnerability to device-related issues	Conservative glycemic targets, caregiver involvement, simplified workflows, frequent reassessment
Mild cognitive impairment	Reduced complexity of insulin adjustments, improved overnight safety, reduced diabetes distress	Risk of incorrect meal announcement, reduced ability to troubleshoot device failures	Repeated education, remote monitoring, caregiver-supported management
Moderate/severe cognitive impairment	Potential reduction of hypoglycemia exposure in selected supervised individuals	Inability to independently manage AID systems, risk of misuse or delayed recognition of technical failures	Use only with reliable caregiver or healthcare staff support, simplified therapeutic goals
Long-term care or nursing-home resident	Remote glucose monitoring, reduction of severe hypoglycemia, potential reduction in hospitalizations	Limited staff expertise, inconsistent device management, institutional workflow barriers	Structured institutional protocols, staff training, telemedicine and caregiver integration

Abbreviations: AID = Automated insulin delivery; TIR = Time in range

Structured education remains a cornerstone of successful AID implementation [24]. Although contemporary systems are highly automated, they still require active user engagement, including meal announcement, infusion set management, recognition of device malfunctions, and interpretation of system alerts. Older adults may require longer training periods, more frequent reinforcement, and simplified educational approaches compared with younger users [7, 50, 51]. Regular reassessment of cognitive and functional abilities should also be incorporated into routine follow-up, particularly in individuals with progressive comorbidities or evidence of cognitive decline [2, 47].

While healthy older adults with preserved functional status may achieve glycaemic targets similar to those recommended for younger adults, less stringent goals remain appropriate for individuals with frailty, significant comorbidities, cognitive impairment, or limited life expectancy [46]. In this context, AID systems should be viewed not as tools for pursuing increasingly aggressive glycaemic control, but rather as instruments that facilitate safer and more individualized diabetes management.

Despite the encouraging evidence currently available, important knowledge gaps remain. Most published studies have enrolled relatively healthy, cognitively intact, and technologically experienced participants. Consequently, the effectiveness and usability of AID systems in frail older adults, individuals with dementia, residents of long-term care facilities, and those with substantial functional limitations remain largely unknown [7, 39, 40].

Further studies are also needed to evaluate outcomes extending beyond traditional glycaemic metrics. Future investigations should assess the impact of AID systems on severe hypoglycaemia-related geriatric-specific outcomes, including frailty trajectories, falls, disability, hospitalizations, preservation of functional status, and caregiver-related outcomes, and quality of life.

From a technological perspective, future generations of AID systems should increasingly incorporate age-friendly design principles. Simplified interfaces, reduced alarm burden, enhanced accessibility for individuals with visual or hearing impairments, remote caregiver monitoring capabilities, and integrated decision-support tools may further improve usability among older adults [52].

## Conclusions

AID systems represent a highly effective and generally safe strategy for managing T1D in older adults, providing clinically meaningful improvements in Time in Range and marked reductions in hypoglycemia risk, which is a primary clinical priority in geriatric care. However, a key distinction must be made between direct evidence and extrapolated data.

Robust RCTs and real-world studies in relatively healthy, independent older adults (≥ 60–65 years) confirm sustained engagement, high system uptake (> 90% automated mode time), and significant glycemic/psychosocial benefits.

Insights regarding complex geriatric management, severe cognitive decline, advanced frailty, and institutional care settings are largely extrapolated from general adult populations or observational geriatric literature. Frail and cognitively impaired individuals remain largely excluded from pivotal AID trials.

Future research must prioritize dedicated studies in vulnerable, frail, and long-term care populations. Clinical implementation in aging T1D populations requires a multidimensional geriatric approach, integrating individualized targets, caregiver involvement, and age-friendly device design to safely bridge current evidence gaps.

## Data Availability

No datasets were generated or analysed during the current study.

## Abbreviations

AID: Automated insulin delivery
HCL: Hybrid closed loop
AHCL: Advanced hybrid closed loop
CL: Closed loop
CGM: Continuous glucose monitoring
SAP: Sensor-augmented pump
MPC: Model predictive control
PID: Proportional-integral-derivative
TIR: Time in range
TBR: Time below range
T1D: Type 1 diabetes
T2D: Type 2 diabetes
ADA: American diabetes association
EASD: European association for the study of diabetes
PROs: Patient-reported outcomes
